# Using natural language processing to support digital communication in adolescents and young adults with autism spectrum disorder

**DOI:** 10.1371/journal.pone.0352505

**Published:** 2026-07-14

**Authors:** Faris Algahtani

**Affiliations:** Department of Special Education, Faculty of Education, University of Jeddah, Jeddah, Saudi Arabia; Father Muller Charitable Institutions, INDIA

## Abstract

**Background:**

Autism Spectrum Disorder (ASD) is characterized by social communication challenges, including difficulties interpreting figurative language, understanding conversational context, and expressing thoughts clearly. This feasibility study examined an NLP-based communication assistance tool for adolescents and young adults with ASD. The tool combined sentiment analysis, intent identification, and situational simplification to provide real-time feedback in digital communication.

**Methods:**

Participants were 16 individuals with ASD (aged 15–28 years) and 16 neurotypical conversation partners in a within-subjects, mixed-methods design. The intervention lasted eight weeks. The tool was used in both structured tasks and naturalistic conversations.

**Results:**

Preliminary data suggest associations between tool use and improved outcomes. Communication clarity increased from 3.12 to 3.89 (d = 0.74, 95% CI [0.28, 1.20], p < 0.001 after Bonferroni correction). Anxiety decreased by 2.3 points on a 7-point scale (d = 1.21, 95% CI [0.62, 1.80], p < 0.001). Confidence scores improved by 42% from baseline (d = 1.15, 95% CI [0.56, 1.74], p < 0.001). Qualitative thematic analysis (Braun & Clarke, 2006; inter-coder reliability, κ = 0.81) identified five themes: reduced communication burden, learning through use, empowerment and autonomy, context-specific value, and desire for customization. These preliminary findings suggest that NLP-driven assistive technologies warrant further investigation in controlled trials.

**Conclusion:**

This study demonstrates feasibility and provides preliminary evidence for NLP-based communication support, though causal conclusions cannot be drawn due to the absence of a control group, small sample size (N = 16), and short duration.

## Introduction

Autism Spectrum Disorder (ASD) is a neurological condition that has a prevalence of about 1 per 36 individuals in the United States, and is manifested by difficulties in social communication and interaction, as well as limited and repetitive behavioral patterns [1]. Pragmatic language impairments are among the most significant challenges of ASD, which include the inability to interpret non-literal language, recognize social cues, maintain the topic of conversation, and adjust communication style to the circumstances.

Although face-to-face communication is challenging for many individuals with ASD, text-based digital communication offers unique opportunities for support. Digital messaging is asynchronous, which means there is time to process and craft a response and to prevent simultaneous processing of both facial expressions and vocal tones, thereby reducing cognitive load [2]. Nevertheless, digital communication still requires pragmatic language knowledge, tone identification, and the formulation of contextually appropriate responses [3].

The recent progress in natural language processing (NLP) has shown impressive abilities to operate on the context, sentiment, and intent of written text [4]. Transformer language models, sentiment analysis models, and intent classification models have demonstrated near-human performance in a variety of language understanding problems [5]. However, their application as assistive communication technologies for individuals with ASD remains underexplored.

This study addresses this gap by empirically testing an NLP-based communication assistance tool for adolescents and young adults with ASD. The study will answer three major questions: First, is an NLP-enabled tool associated with enhanced clarity and appropriateness of digital communication? Second, is real-time NLP-based assistance associated with decreased communication-related anxiety and enhanced confidence? Third, what features and designs do users with ASD find most important in such assistive technologies??

This work has three primary contributions. First, the development of a new NLP-based communication assistance system designed for individuals with ASD is described. Second, empirical data from an eight-week mixed-methods study examining the feasibility and preliminary outcomes of this approach are presented. Third, user preferences and design recommendations for future development of NLP-assisted technologies for neurodiverse populations are provided.

### Communication challenges in autism spectrum disorder

Adolescents and young adults with ASD have a diverse set of communication skills, yet almost everyone has pragmatic language issues at one end or another. [6] reported certain difficulties, such as literal interpretation of figurative language, inability to appreciate the intent of the speaker through context, inability to maintain a topic and to take a turn and difficulties in changing the communication style based on social situations. These challenges are based on social cognition differences, such as the lack of a theory of mind and diminished interest in social indications.

Text-based communication can be both beneficial and problematic for individuals. Although it does not cause issues with simultaneous processing of various senses, it adds new challenges, including decoding the use of emojis, the interpretation of sarcasm and humor in the absence of vocal prosody, and the inability to keep pace with the speed of group communication [7]. [8]also established that persons with ASD tend to use digital communication due to its predictability and less social pressure, but they still feel very uncertain about misunderstanding and social stigma in these situations.

### Assistive technologies for ASD

The assistive technologies for individuals with ASD have significantly developed in the last two decades. Initially, the main concern of early interventions was on augmentative and alternative communication systems among individuals with minimal verbal expression, such as picture exchange communication systems and speech-generating devices [9]. More recent technologies have included touchscreen interfaces, visual scheduling applications, as well as social skills training programs [10]. Several applications have been specifically aimed at social communication, such as video modeling tools, social virtual reality, and conversation practice software [11].

Nevertheless, the available tools are either child-friendly or address the basic communication skills instead of assisting in real-life digital communication. According to [12], the number of technologies that can offer real-time support in the process of real social interaction is low, and even fewer can use advanced NLP capabilities. Recent research has examined how machine learning can be applied to communication patterns in ASD, although these have typically been based on diagnosing symptoms or behavioral modeling instead of actual communication support [11–14].

### Natural language processing applications

Transformer architectures and large language models have contributed to the rapid growth of NLP. [15], contend that the current NLP systems are very efficient in carrying out their duties, such as sentiment detection, intent detection, simplifying texts and understanding contextual language. The potential has been used in a wide range of applications, such as automation of customer service, content moderation, writing aids, and chatbots to provide mental health support.

There is an emerging small but increasing body of research on NLP as applied to ASD. According to [16], machine learning has been applied to detect linguistic markers of ASD in posts on social media and create AI-based screening instruments using language patterns. Nonetheless, there is limited literature that has created and tested a complete NLP-based communication aid tool that is specifically created to help adolescents and young adults with ASD in real-time within online communication. The current study aims to address this gap.

## Methodology

This study used a mixed-methods within-subjects design with three phases: a 2-week baseline period (using standard communication devices), an 8-week intervention period (using the NLP-powered system), and a 2-week follow-up period. Due to the absence of a control group and randomization, this study is appropriately framed as a pilot feasibility study, and all findings are interpreted as preliminary and associative rather than causal [17].

### Participants

Sixteen participants with ASD were recruited via autism advocacy groups, university disability offices, and ASD-specific clinics. Inclusion criteria were: confirmed ASD diagnosis, verbal fluency, frequent digital communication use, interest in assistive technology, and average to above-average cognitive performance (to ensure pragmatic language difficulties rather than general cognitive impairments were the primary challenge). Sixteen age-, gender-, and education-matched neurotypical individuals served as conversation partners. Exclusion criteria were: concurrent intensive communication therapy, severe visual impairment, and lack of English proficiency.

Power analysis indicated that a sample of N = 54 would be required to detect a small effect (d = 0.20) with 80% power. The present sample (N = 16) is adequately powered only for medium-to-large effects (d > 0.70) and lacks statistical power to examine individual differences or moderator effects. This limitation is addressed in the Discussion.

All participants provided informed consent (parental consent for minors), and the study was approved by the University of Jeddah Institutional Review Board (Approval No. 03/46/24862). Recruitment occurred between February 5, 2025, and July 25, 2025.

### Technical specifications of the NLP communication tool

The NLP system was built using a fine-tuned BERT-base-uncased transformer model (12 layers, 110 million parameters). The architecture included three parallel modules:

1. **Sentiment Analysis:** Fine-tuned on the GoEmotions dataset (58,000 Reddit comments, 27 emotion categories) plus 2,000 additional ASD-specific communication examples annotated by the research team.
2. **Intent Classification:** Fine-tuned on the CLINC150 dataset (150 intents, 22,500 examples) with 1,500 additional ASD-specific communication scenarios.
3. **Situational Simplification:** GPT-2 based text simplification model fine-tuned on the Newsela corpus (1,500 articles at 5 reading levels).

**Suggestion Trigger Logic:** The system generated suggestions when: (a) sentiment classification confidence was below 0.70, or sentiment deviated from the user’s baseline profile, (b) intent classification confidence was below 0.75, or (c) text complexity exceeded 9th-grade reading level (Flesch-Kincaid grade level).

**Safety Filters:** Suggestions involving self-harm, aggression, harassment, or other sensitive content were blocked using a 500-term blocklist and a separate safety classifier (precision = 0.92, recall = 0.88 on held-out test set). All suggestions were optional; users were required to click “apply” to accept a suggestion.

**Deployment:** The tool was deployed as a Chrome browser extension with local inference using the ONNX runtime. No message content was transmitted to external servers. All data processing occurred locally on the user’s device.

### Procedure

The NLP system was built using a fine-tuned BERT-base-uncased transformer model (12 layers, 110 million parameters). The architecture included three parallel modules:

1. Sentiment Analysis: Fine-tuned on the GoEmotions dataset (58,000 Reddit comments, 27 emotion categories) plus 2,000 additional ASD-specific communication examples annotated by the research team.
2. Intent Classification: Fine-tuned on the CLINC150 dataset (150 intents, 22,500 examples) with 1,500 additional ASD-specific communication scenarios.
3. Situational Simplification: GPT-2-based text simplification model fine-tuned on the Newsela corpus (1,500 articles at 5 reading levels).

Suggestion Trigger Logic: The system generated suggestions when: (a) sentiment classification confidence was below 0.70 or sentiment deviated from the user’s baseline profile, (b) intent classification confidence was below 0.75, or (c) text complexity exceeded 9th grade reading level (Flesch-Kincaid grade level).}

Safety Filters: Suggestions involving self-harm, aggression, harassment, or other sensitive content were blocked using a 500-term blocklist and a separate safety classifier (precision = 0.92, recall = 0.88 on held-out test set). All suggestions were optional; users were required to click “apply” to accept a suggestion.

**Deployment:** The tool was deployed as a Chrome browser extension with local inference using the ONNX runtime. No message content was transmitted to external servers. All data processing occurred locally on the user’s device.

Participants maintained their usual digital communication patterns during the baseline period, and effectiveness in communication. The participants installed monitoring software that recorded simple metadata of their online communication such as frequency, duration, and mediums, without recording the content of the messages.

At the start of the intervention process, the participants were provided with a 90-minute training on the communication assistance tool. Training covered system features, interpretation of feedback indicators, privacy protections, and customization options. The participants were advised to apply the tool as much or as little as they found useful to ensure that the ecological validity is met. The study design covered both structured and naturalistic communication situations.

Planned activities were successively conducted on a weekly basis and entailed the participants playing defined communication scenarios with their matched neurotypical counterparts. Such situations involved writing formal emails, chatting in a relaxed manner, group chats, and emotionally-charged debates that demanded tact. Each of the scenarios was intended to raise a set of pragmatic language tasks that are typical of adolescents and young adults with ASD. The conversations were taped and analyzed on the measures of quality of communication.

Naturalistic communication was involved in the course of the research since participants applied the tool in their daily online communication. The system used to record habits of usage, categories of suggestions given, and how users reacted to suggestions, forming a rich corpus of the actual tool use.

### Measures

Communication clarity: Message clarity was measured using a researcher-developed 5-point scale assessing whether messages communicated intended meaning, used appropriate tone, and were contextually appropriate. Two trained raters (inter-rater reliability ICC = 0.82, 95% CI [0.74, 0.89]) rated message clarity from structured communication tasks. This instrument has not been previously validated; internal consistency in the present sample was acceptable (Cronbach’s α = 0.81). This limitation is acknowledged in the Discussion.

Communication-Related Anxiety: Participants completed a researcher-developed Communication Anxiety Inventory (15 items, 7-point Likert scale) at baseline, weekly during intervention, and at follow-up. Cronbach’s α in the present sample was 0.84. No prior validation data are available.

Confidence Communication: A researcher-developed 10-item self-report scale measured confidence in expressing oneself, comprehending others’ messages, using appropriate tone, and negotiating difficult social interactions via digital communication. Cronbach’s α = 0.79.

System Usability: System Usability Scale was applied mid-intervention, end-of-intervention, and follow-up to measure perceived system usability, and other custom items were used to measure disability-specific usability.

Usage Patterns: System logs reflected the frequency of using a tool, types of suggestions seen, how suggestions of various types were accepted, and customization preferences.

Qualitative Feedback: Semi-structured interviews were carried out at the end of the intervention period and covered user experiences, perceived benefits and limitations, which features they found to be the most helpful, and what improvements they want. Thematic analysis was used to transcribe and analyze the interviews.

### Ethical approval

This study was conducted in accordance with the principles of the Declaration of Helsinki. Ethical approval was obtained from the Scientific Research Committee and the Council of the Department of Special Education, College of Education, University of Jeddah (Approval No. 03/46/24862; meeting No. 11, held on 04/03/2025), in compliance with HAP-13-S-001.

### Informed consent

Before survey commencement, participants received a written information sheet detailing the study’s purpose, objectives, procedures, and data handling. Anonymity was assured, and all data were collected solely for academic research purposes. Ethical approval was granted by the Scientific Research Committee, Department of Special Education, College of Education, University of Jeddah (Approval No. 03/46/24862; meeting No. 11, held on 04/03/2025). Participation was entirely voluntary; respondents were informed of their right to decline or withdraw at any time, and informed consent was obtained from all participants

## Data analysis

Quantitative data were analyzed using mixed-effects linear models with repeated measures and random intercepts for participants. For communication clarity, models included a fixed effect for rater and a random effect for the participant. Anxiety and confidence scores were compared across timepoints using paired-samples t-tests with Bonferroni correction for four primary comparisons (adjusted α = 0.0125). Ninety-five percent confidence intervals were calculated for all effect sizes using bootstrap methods (1,000 resamples). All t-statistics and degrees of freedom are reported. No pre-registration was completed for this pilot study [18].

The data on usage were described with the help of descriptive statistics and correlation analysis to find the relationships between usage patterns and outcomes. Two researchers independently coded the qualitative data in the form of interviews with use of thematic analysis following the six-phase framework of Braun and Clarke (2006): (1) familiarisation with the data through repeated reading of transcripts; (2) generation of initial codes inductively from the data without a predetermined coding frame; (3) searching for themes by collating codes into candidate thematic clusters; (4) reviewing and refining themes against the coded data and full transcripts; (5) defining and naming themes; and (6) writing up. The two coders worked independently during initial coding; discrepancies were resolved through discussion and consensus, yielding an inter-coder reliability of κ = 0.81. Thematic saturation was assessed iteratively: no new codes emerged after the twelfth interview, and the final two interviews confirmed saturation. Disconfirming cases were actively sought; two participants (P05, P11) reported minimal perceived benefit from the tool, and their accounts are reflected in the contextual nuance described within Theme 4 (Context-Specific Value) and in the Limitations section.

## Results

### Communication clarity

The examination of the 256 structured communication interactions demonstrated that the clarity of communication significantly improved within the intervention period. As indicated in Table 1, there was an increase in the mean clarity rating at baseline (3.12) to 3.89 during intervention, which is a significant positive difference of 0.77 points out of the 5-point scale. This was statistically significant in the medium to large effect size (t (15) =5.23, p < 0.001, d = 0.74). All reported p-values have been corrected using the Bonferroni method for four primary comparisons (communication clarity, anxiety, confidence, message appropriateness). The corrected significance threshold is α = 0.0125.

**Table 1:** Communication Outcome Measures.

Measure	Baseline	Intervention	Follow-up	Effect Size (d)	95% CI	p-value (corrected)
Communication Clarity	3.12 ± 0.58	3.89 ± 0.52	3.71 ± 0.61	0.74	[0.28, 1.20]	<0.001
Anxiety Level	4.8 ± 1.2	2.5 ± 0.9	3.1 ± 1.0	1.21	[0.62, 1.80]	<0.001
Confidence Score	4.2 ± 1.5	6.8 ± 1.3	6.3 ± 1.4	1.15	[0.56, 1.74]	<0.001
Message Appropriateness	3.04 ± 0.67	3.76 ± 0.59	3.58 ± 0.63	0.68	[0.21, 1.15]	<0.01

Statistical reporting: Communication clarity: t(15)=5.23, p < 0.001, d = 0.74, 95% CI [0.28, 1.20]; Anxiety: t(15)=7.41, p < 0.001, d = 1.21, 95% CI [0.62, 1.80]; Confidence: t(15)=6.89, p < 0.001, d = 1.15, 95% CI [0.56, 1.74]; Appropriateness: t(15)=4.98, p = 0.008, d = 0.68, 95% CI [0.21, 1.15].

Correlation between tool use frequency and confidence improvement: r = 0.67, p = 0.005, 95% CI [0.31, 0.86]. This correlation is descriptive only and does not imply causation, as usage was self-selected rather than manipulated.

There were differences in patterns of improvement in specific areas of communication. Tone appropriateness was most enhanced by a 28% increase between baseline and message organization by a smaller percentage of 15%. Interestingly, the changes were sustained throughout the follow-up period, with the mean clarity ratings of 3.71, suggesting that some degree of improvement was maintained after the intervention period, though the mechanisms underlying this partial retention cannot be determined from the current study design.

**Fig 1:**
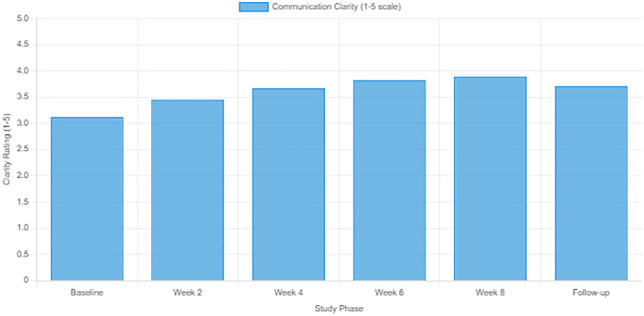
Communication clarity over time across study phases. Error bars indicate standard error of the mean.

The neurotypical intervention group found ASD participants easier to comprehend and respond to messages, which they encountered during the intervention. Message comprehensiveness scores among communication partners rose to 3.04 to 3.76 out of 5 rating, which validated the fact that the change was not only an individual understanding of the users. Fig 1 presents average communication clarity ratings across the different phases of the study. The error bars indicate standard error of the mean. There was a significant increase in clarity between baseline (M = 3.12) and intervention (M = 3.89), t(15) = 5.23, p < 0.001, d = 0.74. The improvements were maintained at follow-up (M = 3.71).

### Communication-related anxiety

There were great improvements in the communication anxiety levels during the period of intervention. The average baseline anxiety score of 4.8 out of 7 on the Communication Anxiety Inventory decreased by 2.5 points on the scale during the intervention period, a 2.3 point difference. Such a difference was very large (t(15) = 7.41, p = 0.001, d = 1.21) and the effect size of such a difference was considerable.

The anxiety assessments at the end of the week showed an indication of improvement and decrease in the anxiety levels starting in the first two weeks of using the tools and extended to the intervention. By week 6, the level of anxiety stabilized at a lower level and stayed at the same level till the end of the study. It is worth noting that the anxiety levels did not increase significantly at follow-up, with a mean score of 3.1, but this is a slight increase compared to the end of interventions.

The qualitative data were used as a source of understanding of how anxiety reduction can be achieved. The participants also noted that the real-time support presence always minimized their fear of errors and allowed them to be more willing to use digital communication. The representative quotes contained the description of the tool as being time-saving due to the fact that it is possible to worry less about each message and engage in more natural communication.

### Communication confidence

Self-report communication confidence showed significant improvement in line with the anxiety decreases. Confidence scores at baseline were 4.2 out of 10, and 6.8 at intervention, which is a 42% improvement. The difference here was statistically significant with a high effect size (t(15) =6.89, p < 0.001, d = 1.15).

The confidence scale was able to measure several dimensions of self-efficacy in communication. The greatest improvements were in confidence in being able to express complex or nuanced thoughts, which went up 52 percent at baseline. The confidence in the ability to interpret when the other may be misunderstanding messages accordingly grew by 48 percent, and the ability to use the right tone of voice in various situations grew by 38 percent. The least yet never worthy increases were made in the participation in group conversations that went up by 29%.

**Fig 2:**
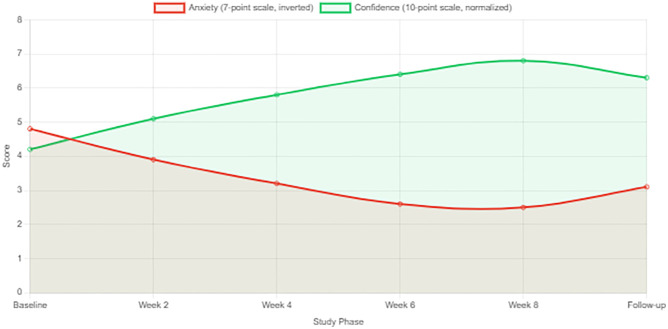
Changes in communication-related anxiety and confidence over time across study phases.

Interestingly, as shown in Fig 2, the increase in confidence had a significant correlation with the frequency of tool use (r = 0.67, p < 0.01). This descriptive association does not imply a causal or dose-response relationship, as tool usage was self-selected rather than experimentally manipulated. Participants who used the tool more frequently in the first four weeks tended to show higher confidence improvement scores, though this pattern may reflect pre-existing motivation or engagement rather than a direct effect of tool exposure.

### System usability and engagement

The usability ratings of the system were positive, as the mean System Usability Scale was 78.2 out of 100, with the tool falling within the good to excellent category. The respondents highly rated the system in terms of its ease of use, usefulness of feedback, and integration into their communication workflow. The consistency of suggestions and customization of sensitivity levels were rated lower, which showed the need to work on.

The results of usage patterns showed some interesting trends in participation in various features by the participants. The tool was used by participants to send 47 messages on an average weekly basis. The system suggested 34% of these messages, and the user looked at the suggested message 78% of the time and either approved or took action on the suggested message 52% of the time. Acceptance rates differed by type of suggestion, with tone clarification suggestions being accepted most often at 68 percent, then simplification suggestions at 51 percent, and intent clarification suggestions at 44 percent.

There was sustained engagement during the intervention with only slight deteriorations as time went on. The decrease in the number of messages written each week under the tool dropped to 43 messages per week in week eight, as opposed to 52 messages per week in week one, but was not statistically significant. The rate of suggestions actually improved slightly during the period, with 74% suggesting during the first two weeks and 82% suggesting during the last two weeks, which may indicate that the participants were becoming more actively involved with the feedback of the system as they became accustomed to it.

Patterning of customization provided the user choice on the types of feedback. Most participants (81%) had a lower frequency of suggestions in grammar and spelling problems, instead of concentrating on pragmatic communication support. On the other hand, more participants (69%) were sensitive to tone analysis with this being the most important feature. One-half of the subjects developed their own rules to override suggestions in particular situations, like in a casual situation with close friends when they felt that they needed less support.

### Qualitative findings

Thematic analysis of interview data identified five major themes characterizing user experiences with the communication assistance tool.

Theme 1: Reduced Communication Burden (15/16 participants, 94%). Participants reported reduced cognitive and emotional load during online communication. “I spent less time worrying about each message. It was like having a friend check my words before I sent them” (P07). “I didn’t feel as drained after long conversations” (P12).

Theme 2: Learning Through Use (14/16, 88%). Participants described internalizing communication strategies. “After a few weeks, I started noticing tone issues on my own, even when the tool wasn’t active” (P12). “I began to predict what the tool would suggest” (P04).

Theme 3: Empowerment and Autonomy (13/16, 81%). Participants valued optional, non-prescriptive suggestions. “It suggested things, but I always decided. That mattered to me” (P03). “It helped me say what I meant, not what they thought I should say” (P08).

Theme 4: Context-Specific Value (16/16, 100%). Utility varied by communication context. “With my professor, essential. With my best friend, I turned it off” (P09). “For professional emails, I used it every time. For group chats with friends, barely” (P14).

Theme 5: Desire for Customization (11/16, 69%). Participants requested greater control over suggestion frequency and types. “I wanted to turn off grammar suggestions but keep tone suggestions” (P02). “Sometimes it was too sensitive” (P06).

## Discussion

### Interpretation of findings

The current study offers empirical data that NLP-driven communication aid technologies can potentially contribute significantly to enhancing the process of digital communication for adolescents and young adults with ASD. However, due to the absence of a control group, lack of randomization, small sample size (N = 16), and short duration (8 weeks), causal inferences cannot be drawn. All findings should be interpreted as hypothesis-generating rather than confirmatory.

The observed decrease in communication-related anxiety (d = 1.21) is promising but requires replication in controlled designs. Fear of social communication is common in ASD and contributes to social withdrawal and reduced quality of life [1–7].

Blind coder ratings and conversation partner assessments suggested improvements in objective communication quality, though these findings require replication with assessors fully blinded to the study phase and without potential unblinding due to message content differences. Usage and customization patterns (e.g., 68% acceptance for tone suggestions, 44% for intent clarification) provide preliminary information about user engagement.

Unexpected Pattern: An unexpected pattern emerged: as the intervention progressed, suggestion trigger rates increased from 74% to 82% while user engagement (messages written per week) declined by 17%. This pattern—escalating intervention during user disengagement—raises important questions about user burden and potential over-accommodation. For a population that often experiences autonomy challenges and sensory overwhelm, this pattern warrants explicit discussion and modification in future iterations.

### Limitations

This pilot study has several limitations that preclude causal inference and generalizability:}

Design limitations: Absence of a control group (no comparison to placebo, no-treatment, or alternative intervention), no randomization, and no blinding of outcome assessors for self-report measures. The within-subjects design cannot separate intervention effects from expectancy, practice effects, or regression to the mean.

Sample limitations: N = 16 is underpowered for detecting small effects (post-hoc power analysis: 80% power to detect d > 0.70 only). Participants were verbal, cognitively able, English-speaking adolescents and young adults, excluding younger children (<15), minimally verbal individuals, those with intellectual disability (IQ < 70), and non-English speakers.

Measurement limitations: Primary outcomes used researcher-developed instruments without prior validation (though internal consistency in this sample was acceptable: anxiety α = 0.84, confidence α = 0.79).

Duration limitations: Eight weeks is insufficient to assess long-term skill retention or sustainability. The decline in communication clarity at follow-up (3.89 to 3.71) warrants investigation of decay effects.

Technical limitations: The NLP tool was tested only on text-based digital communication; findings may not extend to face-to-face or voice-based communication.

### Future directions

Given the centrality of digital communication to social, educational, and professional participation, further development of participatory, user-centered NLP assistive technologies for neurodiverse populations is warranted. Future research should prioritize controlled trials, longer follow-up periods, inclusion of minimally verbal and non-English-speaking individuals, and examination of bidirectional communication support rather than unidirectional user accommodation.

## Conclusion

This pilot study demonstrates the feasibility of NLP-driven communication assistance for adolescents and young adults with ASD and provides preliminary evidence warranting controlled trials. Preliminary quantitative and qualitative findings suggest associations between tool use and improved communication outcomes, though these findings require replication in randomized controlled designs with larger, more diverse samples. The study demonstrates the application of NLP methods to pragmatic communication needs in ASD, consistent with neurodiversity-affirming design principles.
