# Supplementary material for: Using natural language processing to support digital communication in adolescents and young adults with autism spectrum disorder
Source: PLoS One. 2026 Jul 14;21(7):e0352505. doi: 10.1371/journal.pone.0352505 (PMC13367686; doi:10.1371/journal.pone.0352505)
Supplement: Attachment — Submitted filename: RESPONSE TO THE REVIEWERS (1).docx [file pone.0352505.s002.docx]

**RESPONSE TO REVIEWERS**

**Manuscript Title:** Using Natural Language Processing to Support Digital Communication in Adolescents and Young Adults with Autism Spectrum Disorder: A Pilot Feasibility Study

Dear Academic Editor Dr. Ramandeep Kaur and Reviewers,

Thank you for your thoughtful and constructive reviews of my manuscript. I appreciate the recognition that the topic is timely and important, and I have carefully considered all comments. I have substantially revised the manuscript to address the methodological, statistical, transparency, and reproducibility concerns raised.

Below I provide a point-by-point response to all editor and reviewer comments.

**RESPONSE TO ACADEMIC EDITOR (Dr. Ramandeep Kaur)**

**Comment 1 (Statistical rigor):** *The current statistical analysis is insufficient. There is lack of correction for multiple comparisons, absence of confidence intervals, and limited reporting of test statistics. The interpretation of findings (e.g., "dose-response") is not justified given the study design.*

**Response:** I have substantially revised the statistical analysis. Specifically:

- I added **Bonferroni correction** for four primary comparisons (communication clarity, anxiety, confidence, message appropriateness), with adjusted α = 0.0125.
- I added **95% confidence intervals** for all effect sizes using bootstrap methods (1,000 resamples).
- I added **complete test statistics** (t-values, degrees of freedom) for all primary outcomes.
- I removed the **"dose-response" causal language** and replaced it with descriptive correlation (r = 0.67, p = 0.005, 95% CI [0.31, 0.86]) with an explicit statement that this is descriptive only and does not imply causation.

**Comment 2 (Study design limitations):** *The absence of a control group, small sample size (N=16), and short intervention duration significantly limit causal inference and generalizability. The manuscript must clearly position the study as a pilot/feasibility study and temper all causal claims.*

**Response:** I agree entirely. The following changes have been made:

- **Title revised** to include "A Pilot Feasibility Study"
- **Abstract revised** to frame findings as "preliminary" and "associative rather than causal"
- **Methods section** now explicitly states: "Due to the absence of a control group and randomization, this study is appropriately framed as a pilot feasibility study, and all findings are interpreted as preliminary and associative rather than causal."
- **Power analysis added** (N=54 required for small effects; N=16 powered only for medium-to-large effects)
- **Conclusion entirely rewritten** to reflect preliminary, hypothesis-generating nature of findings
- All causal verbs (e.g., "improves," "demonstrates effectiveness") replaced with associative language (e.g., "is associated with," "suggests," "warrants further investigation")

**Comment 3 (Data availability and reproducibility):** *There is inconsistency regarding data availability across reviews. The manuscript must explicitly clarify what data, code, and materials are available.*

**Response:** I have completely revised the Data Availability Statement to comply with PLOS policy:

- Data are available upon request to the **University of Jeddah Data Access Committee** (datacommittee@uj.edu.sa), a non-author institutional contact
- De-identified data will be provided under a data transfer agreement for replication purposes
- **NLP tool code and model weights** will be made available at a GitHub repository (anonymized URL provided for review; to be made public upon acceptance)
- I have clarified that the tool has not been open-sourced at the time of submission but will be upon publication

**Comment 4 (Outcome measures – non-validated instruments):** *The use of researcher-developed tools without psychometric validation weakens interpretability.*

**Response:** I have:

- Explicitly acknowledged that primary outcomes used **researcher-developed instruments without prior validation**
- Added **Cronbach's α** for each measure from the present sample (anxiety: α = 0.84; confidence: α = 0.79; clarity: α = 0.81)
- Added this as a **limitation** in the Discussion section

**Comment 5 (Qualitative analysis):** *Reporting is below accepted standards. Include methodology, participant quotes, theme frequencies, and reliability measures.*

**Response:** I have completely rewritten the qualitative analysis section to meet Braun & Clarke (2006) standards, including:

- **Coding methodology** (six phases of thematic analysis)
- **Inter-coder reliability** (κ = 0.81, 95% CI [0.74, 0.88])
- **Saturation assessment** (achieved after 12 interviews)
- **Theme frequencies** (reported as n/N, percentage for each theme)
- **Representative participant quotes** (with participant IDs)
- **Disconfirming cases** (three participants who found the tool intrusive)

**Comment 6 (Conclusions overstated):** *The conclusions must be revised to reflect limitations. Given the small sample size, short duration, and lack of control group, the study should be framed as preliminary or pilot work.*

**Response:** The Conclusion section has been entirely rewritten. It now:

- Opens with: "This pilot study demonstrates the feasibility... and provides preliminary evidence warranting controlled trials"
- Explicitly states: "causal inferences cannot be drawn"
- Calls for randomized controlled trials with larger, more diverse samples
- Removes all definitive claims of effectiveness

**RESPONSE TO REVIEWER #1 (Dr. Rinku Sharma Dixit)**

**Reviewer comment:** *The claims are stronger than the study design can support. The paper presents the work as evidence that the NLP tool improves communication, reduces anxiety, and increases confidence, but the study appears to use a small within-subject sample without a randomized design or control group.*

**Response:** I have substantially tempered all claims throughout the manuscript. Key changes are summarized below:

| Original claim | Revised claim |
| --- | --- |
| "The tool improves communication" | "Tool use is associated with improved communication outcomes (preliminary)" |
| "This paper has shown that NLP systems could be helpful" | "This pilot study demonstrates feasibility and provides preliminary evidence" |
| "Proves the effectiveness of this method" | "Suggests associations that warrant further investigation" |

**Reviewer comment:** *There is a mismatch between the framing of the paper and the sample studied. The title, abstract, and conclusions repeatedly refer to "children with autism," but the participant age range is reported as 15-28 years.*

**Response:** I have corrected this throughout. The title now reads "Adolescents and Young Adults" rather than "Children." All instances of "children" have been changed to "adolescents and young adults" or "individuals with ASD" as appropriate.

**Reviewer comment:** *The description of the intervention is not sufficiently detailed for technical evaluation or reproducibility. The manuscript does not explain what models were used, what data they were trained on, how outputs were generated, what safeguards were in place.*

**Response:** I have added a new subsection titled **"Technical Specifications of the NLP Communication Tool"** that includes:

- Model architecture: BERT-base-uncased (12 layers, 110M parameters)
- Training data: GoEmotions (58k examples), CLINC150 (22.5k examples), Newsela corpus (1,500 articles), plus ASD-specific examples (2,000-1,500 per module)
- Suggestion trigger logic (sentiment confidence <0.70, intent confidence <0.75, reading level >9th grade)
- Safety filters: 500-term blocklist + safety classifier (precision=0.92, recall=0.88)
- Deployment: Chrome extension, local ONNX runtime, no external data transmission

**Reviewer comment:** *The statistical analysis is underreported. Confidence intervals are not reported. It is unclear how Bonferroni correction was applied.*

**Response:** See my response to Editor Comment 1 above. All requested statistical details have been added.

**Reviewer comment:** *Outcome measurement needs clarification. Several key outcomes rely on self-report, which should be discussed more explicitly as a limitation.*

**Response:** I have:

- Explicitly identified which measures are self-report (anxiety, confidence) vs. observer-rated (clarity)
- Added self-report as a limitation in the Discussion
- Added internal consistency (Cronbach's α) for all measures

**Reviewer comment:** *The study timeline is described in slightly different ways across sections, and the data-availability statements appear contradictory.*

**Response:** I have:

- Standardized the timeline description across all sections
- Resolved the data availability contradiction (see response to Editor Comment 3)
- Ensured consistency between online submission form and manuscript

**Reviewer comment:** *The manuscript would benefit from major language editing.*

**Response:** The manuscript has been professionally copy-edited. Changes are visible in the tracked changes version.

**RESPONSE TO REVIEWER #2 (Adrian C Demidont)**

**Reviewer comment:** *No datasets, model code, weights, or instrument documentation accompany the submission which provides no opportunity to reproduce results.*

**Response:** I acknowledge this limitation. The following actions have been taken:

- I added a statement that **code and model weights will be made publicly available** upon acceptance at GitHub (anonymized URL provided)
- I added **detailed technical specifications** to enable independent reimplementation
- Data will be available upon request to the institutional Data Access Committee

**Reviewer comment:** *With N = 16 over 8 weeks, the study is underpowered. The tool generated rich continuous process data — messages sent, suggestions triggered, viewed, accepted — reported only as full-period aggregates.*

**Response:** I have:

- Added **power analysis** acknowledging that N=16 is only powered for medium-to-large effects
- **Added new Figure 3** (in revised manuscript; not shown in text version) displaying weekly usage trends for all metrics
- Added temporal trajectory discussion in Results

**Reviewer comment:** *"Transformer model" describes thousands of architectures. Without architecture, training corpus, fine-tuning procedure, prompt strategy, deployment method, and safety filters, reviewers cannot evaluate what was tested.*

**Response:** See my response to Reviewer #1 on technical specifications. All requested details have been added in the new Technical Specifications subsection.

**Reviewer comment:** *Four paired t-tests are reported without correction for multiple comparisons (family-wise error ≈ 19%), confidence intervals, control group, or blinding.*

**Response:** See my response to Editor Comment 1. Bonferroni correction (α = 0.0125) and 95% CIs have been added. Lack of control group and blinding are now explicitly listed as limitations.

**Reviewer comment:** *A usage–confidence correlation (r = 0.67) is described as "dose-response," but dose was self-selected rather than manipulated, leaving causation unclear.*

**Response:** I have removed all "dose-response" language. The correlation is now reported as descriptive, with the explicit caveat: "This correlation is descriptive only and does not imply causation, as usage was self-selected rather than manipulated."

**Reviewer comment:** *Communication Clarity declined from 3.89 to 3.71 at follow-up but is described as "internalization"; anxiety rebounded by 24% but is described as "maintained."*

**Response:** I have revised these interpretations to be more accurate and less positive:

- Follow-up decline is now described as "remained above baseline but decreased from intervention peak" with explicit acknowledgment that "decline at follow-up warrants investigation of decay effects"
- Anxiety rebound is now described accurately, with the follow-up increase noted

**Reviewer comment:** *The Conclusion's "this paper has shown" reads as overstated. The statistical analysis of this uncontrolled study does not provide mathematically interpretable benefits sufficient to support causal claims.*

**Response:** The Conclusion has been completely rewritten. It now:

- Opens with "This pilot study demonstrates feasibility... and provides preliminary evidence"
- Explicitly states that causal claims cannot be made
- Calls for controlled trials

**Reviewer comment:** *The suggestion trigger rate rose from 74% to 82% while engagement declined 17% — the tool escalated intervention as users disengaged. For a population that often experiences autonomy challenges and sensory overwhelm, this pattern deserves explicit discussion.*

**Response:** I have added a new paragraph in the Discussion explicitly addressing this unexpected pattern

"An unexpected pattern emerged: as the intervention progressed, suggestion trigger rates increased from 74% to 82% while user engagement (messages written per week) declined by 17%. This pattern—escalating intervention during user disengagement—raises important questions about user burden and potential over-accommodation. For a population that often experiences autonomy challenges and sensory overwhelm, this pattern warrants explicit discussion and modification in future iterations."

**Reviewer comment:** *The sample excludes minimally verbal individuals and those with intellectual disability — the subpopulations most likely to need assistive technology.*

**Response:** I have:

- Added this as a **key limitation** in the expanded Limitations section
- Added to **Future Directions** as a priority for subsequent research

**Reviewer comment:** *Five positively valenced themes are reported without participant quotes, counts per theme, coding methodology, inter-coder reliability, or disconfirming cases — below the standards described by Braun & Clarke (2006).*

**Response:** See my response to Editor Comment 5. The qualitative analysis has been completely rewritten to meet Braun & Clarke standards, including all requested elements.

**Reviewer comment:** *Context-dependent utility and user-generated override rules are valuable findings that are currently buried.*

**Response:** I agree. These findings have been:

- Elevated to **prominent positions** in the Results
- Highlighted in the **Discussion** as key insights for future design
- Moved earlier in the Results section

**RESPONSE TO REVIEWER #3 (Dr. A Ajina)**

**Reviewer comment:** *The limited sample size and generalizability, as only 16 verbal and cognitively able teenagers and young adults with ASD participated, making it difficult to draw conclusions for younger children, minimally verbal individuals, or those with intellectual disabilities.*

**Response:** I have addressed this by:

- Explicitly listing excluded populations in Limitations
- Adding a table specifying generalizability boundaries
- Framing the study as a pilot that cannot generalize to excluded populations

**Reviewer comment:** *The study's short eight-week duration means that long-term effects and skill retention are unknown.*

**Response:** I added this as a primary limitation. I also added explicit mention of the follow-up decline (3.89 to 3.71) as evidence that decay effects warrant investigation.

**Reviewer comment:** *The tool was found to be less useful in informal communication contexts. Some participants desired more customization.*

**Response:** These are now highlighted as key findings:

- Theme 4 (Context-Specific Value) now prominently features the finding that utility varies by context
- Theme 5 (Desire for Customization) captures customization requests
- The Discussion section notes that the tool was most valued in high-stakes, unfamiliar contexts

**Reviewer comment:** *The research was limited to English-speaking participants, excluding non-English speakers who might benefit from such technology.*

**Response:** I added this to Limitations and Future Directions as a priority for subsequent research.

**Reviewer comment:** *The data cannot be shared publicly due to ethical reasons, which may hinder external validation or replication of the findings.*

**Response:** See my response to Editor Comment 3. I have provided a clear pathway for data access via the institutional Data Access Committee, and code/model weights will be made public upon acceptance.
