# Supplementary material for: Using natural language processing to support digital communication in adolescents and young adults with autism spectrum disorder
Source: PLoS One. 2026 Jul 14;21(7):e0352505. doi: 10.1371/journal.pone.0352505 (PMC13367686; doi:10.1371/journal.pone.0352505)
Supplement: Attachment — Submitted filename: Response to Reviewer .docx [file pone.0352505.s003.docx]

**Response to Reviewers**

**Dear Editor,**

Thank you for your continued evaluation of our manuscript and for the constructive feedback provided by you and the reviewers.

I appreciate the opportunity to submit a second revised version of our manuscript. We have carefully addressed all comments and concerns raised in the latest review round. In particular, we have revised the manuscript to improve consistency in terminology, clarified the interpretation of findings, resolved the data availability statements, and expanded the description of the qualitative analysis procedures. We have also conducted a thorough review of the manuscript to ensure consistency between the response letter and the revised text.

A detailed point-by-point response to all comments is provided in the accompanying “Response to Reviewers” document, and all modifications have been highlighted in the tracked-changes version of the manuscript.

I am grateful for the reviewers’ and editor’s valuable suggestions, which have helped us improve the quality, clarity, and transparency of our work. We hope that the revised manuscript is now suitable for publication in PLOS ONE.

**Thank you for your time and consideration.**

Manuscript Title: Using Natural Language Processing to Support Digital Communication in Adolescents and Young Adults with Autism Spectrum Disorder: A Pilot Feasibility Study

Journal: PLOS ONE

Date: June 3, 2026

Dear Dr. Kaur and Reviewers,

We sincerely thank the Academic Editor and reviewers for their thorough and constructive feedback on our revised manuscript. We have carefully addressed each point raised and believe the manuscript is substantially improved as a result. Below, we provide detailed responses to each concern, with corresponding changes clearly indicated in the tracked-changes version of the manuscript.

**RESPONSES TO ACADEMIC EDITOR COMMENTS**

**Reviewer Comment:** *Although the authors indicate that all references to 'children with autism' were replaced with 'adolescents and young adults,' multiple sections of the manuscript continue to refer to children with ASD despite the participant age range being 15–28 years.*

**Response:** I apologize for these oversights. I have now conducted a comprehensive review of the entire manuscript and corrected all remaining instances.

**Reviewer Comment:** *The response letter states that all 'dose-response' language has been removed; however, the Results section continues to describe the correlation between tool use and confidence improvement as indicating a 'dose-response relationship.'*

**Response:** I thank the editor for identifying this persistent inconsistency. The sentence in the Confidence section has been revised. The revised text now reads: 'the increase in confidence had a significant correlation with the frequency of tool use (r = 0.67, p < 0.01). This descriptive association does not imply a causal or dose-response relationship, as tool usage was self-selected rather than experimentally manipulated. Participants who used the tool more frequently in the first four weeks tended to show higher confidence improvement scores, though this pattern may reflect pre-existing motivation or engagement rather than a direct effect of tool exposure.' This revision removes causal language and explicitly contextualises the finding within the observational nature of the study.

**Reviewer Comment:** *Concerns regarding overinterpretation of follow-up findings have not been fully resolved. The manuscript continues to suggest that participants 'internalized communication strategies' despite observed declines between intervention and follow-up phases.*

**Response:** I agree that this language implied a mechanistic interpretation not supported by the study design. The phrase 'which indicates that the participants internalized communication strategies acquired in the course of using the tools' has been replaced with 'suggesting that some degree of improvement was maintained after the intervention period, though the mechanisms underlying this partial retention cannot be determined from the current study design.' This revision describes the observed pattern without attributing it to a specific process.

**Reviewer Comment:** *Data availability statements remain inconsistent across the submission. Different sections alternatively indicate that data are fully available without restriction, unavailable due to ethical considerations, available upon request from authors, or available through a Data Access Committee.*

**Response:** I recognise that the previous Data Availability section was internally inconsistent and ambiguous. I have replaced it with a single, coherent statement that is compliant with PLOS ONE data-sharing requirements. The revised statement reads: 'The data underlying this study are not publicly available due to ethical restrictions imposed by the University of Jeddah Institutional Review Board (Approval No. 03/46/24862) and participant consent agreements, which did not include provision for public data sharing. Qualified researchers may submit a formal request for access to de-identified data to the University of Jeddah Data Access Committee (datacommittee@uj.edu.sa); requests will be reviewed and, if approved, data will be shared under a data transfer agreement. The NLP tool source code and model weights will be made publicly available upon acceptance of this manuscript via a repository currently blinded for peer review.' This replaces all prior inconsistent statements.

**Reviewer Comment:** *The qualitative analysis has been improved through the inclusion of participant quotes and theme frequencies; however, several elements described in the response letter are not clearly evident in the manuscript. The coding procedure, thematic analysis process, saturation procedures, and disconfirming cases should be described more explicitly.*

**Response:** I have substantially expanded the qualitative methods description in the Data Analysis section. The revised text now explicitly describes the six-phase Braun and Clarke (2006) framework used, the inductive coding approach, the inter-coder reliability procedure (kappa = 0.81), the iterative assessment of thematic saturation (confirmed after the twelfth interview, with the final two confirming saturation), and the active search for disconfirming cases. Specifically, participants P05 and P11 reported minimal perceived benefit from the tool; their accounts are reflected in Theme 4 (Context-Specific Value) and the Limitations section. All additions are marked in the tracked-changes manuscript.

**Reviewer Comment:** *The manuscript still requires careful language editing. Numerous grammatical inconsistencies, duplicated content, formatting issues, and awkwardly constructed sentences remain.*

**Response:** I have conducted a thorough editorial review of the full manuscript, correcting grammatical inconsistencies, removing duplicated content (including the duplicated Technical Specifications/Procedure section), reformatting irregularities, and revising awkwardly phrased sentences. All changes are visible in the tracked-changes version.

**Reviewer Comment:** *The response letter indicates that additional analyses and figures were added (e.g., weekly usage trends), but these additions are not clearly identifiable within the revised manuscript.*

**Response:** I apologise for the lack of clarity in the previous revision. In this revision, I have ensured that all additions described in the response letter are identifiable within the manuscript text and are explicitly referenced where appropriate. Tracked changes clearly mark all new content.

Sincerely,

Prof. Faris

Corresponding Author
