# Supplementary material for: Using natural language processing to support digital communication in adolescents and young adults with autism spectrum disorder
Source: PLoS One. 2026 Jul 14;21(7):e0352505. doi: 10.1371/journal.pone.0352505 (PMC13367686; doi:10.1371/journal.pone.0352505)
Supplement: Peer review history

# Peer review: Decision Letter 0

**DOI:** 10.1371/journal.pone.0352505.r001

**Authors:** Ramandeep Kaur

7 May 2026

PONE-D-26-04125

Using Natural Language Processing to Improve Communication Tools for Children with Autism Spectrum Disorders

PLOS One

Dear Dr. ALGAHTANI,

Thank you for submitting your manuscript to PLOS ONE. After careful consideration, we feel that it has merit but does not fully meet PLOS ONE’s publication criteria as it currently stands. Therefore, we invite you to submit a revised version of the manuscript that addresses the points raised during the review process.

==============================

The manuscript addresses a relevant and timely topic; however, it currently falls short of PLOS ONE’s publication criteria in terms of methodological rigor, transparency, and reproducibility.

**Changes required for acceptance:**

- The statistical analysis needs to be strengthened with appropriate reporting (including test statistics, confidence intervals, and correction for multiple comparisons where applicable). Interpretations must be aligned with the study design, avoiding causal claims in the absence of controlled comparisons.
- The description of the NLP tool is insufficient. Clear details regarding model architecture, training data, implementation, and functioning are required to ensure transparency and allow reproducibility.
- Data availability must be clearly stated in accordance with PLOS policy. Any restrictions should be explicitly justified, and access to underlying data and materials should be clarified.
- Outcome measures require clarification, particularly if non-validated or researcher-developed instruments are used. Their limitations must be explicitly acknowledged.
- The qualitative analysis needs to meet accepted reporting standards, including description of methodology, coding process, and inclusion of representative data (e.g., participant quotes).
- The conclusions must be revised to reflect the limitations of the study design. Given the small sample size, short duration, and lack of control group, the study should be framed as preliminary or pilot work rather than providing definitive evidence of effectiveness.

**Recommended improvements:**

- Provide more detailed reporting of usage and engagement data over time to strengthen interpretation.
- Expand the discussion on context-specific utility and user adaptation patterns, which appear to be meaningful findings.
- Address limitations related to sample characteristics and generalizability more explicitly.
- Ensure consistency between reported results and their interpretation, particularly in follow-up findings.

==============================

Please submit your revised manuscript by Jun 21 2026 11:59PM. If you will need more time than this to complete your revisions, please reply to this message or contact the journal office at plosone@plos.org. When you're ready to submit your revision, log on to https://www.editorialmanager.com/pone/ and select the 'Submissions Needing Revision' folder to locate your manuscript file.

Please include the following items when submitting your revised manuscript:

- A letter that responds to each point raised by the academic editor and reviewer(s). You should upload this letter as a separate file labeled 'Response to Reviewers'.
- A marked-up copy of your manuscript that highlights changes made to the original version. You should upload this as a separate file labeled 'Revised Manuscript with Track Changes'.
- An unmarked version of your revised paper without tracked changes. You should upload this as a separate file labeled 'Manuscript'.

If you would like to make changes to your financial disclosure, please include your updated statement in your cover letter. Guidelines for resubmitting your figure files are available below the reviewer comments at the end of this letter.

If applicable, we recommend that you deposit your laboratory protocols in protocols.io to enhance the reproducibility of your results. Protocols.io assigns your protocol its own identifier (DOI) so that it can be cited independently in the future. For instructions see: https://journals.plos.org/plosone/s/submission-guidelines#loc-laboratory-protocols. Additionally, PLOS ONE offers an option for publishing peer-reviewed Lab Protocol articles, which describe protocols hosted on protocols.io. Read more information on sharing protocols at https://plos.org/protocols?utm_medium=editorial-email&utm_source=authorletters&utm_campaign=protocols.

As the corresponding author, your ORCID iD is verified in the submission system and will appear in the published article. PLOS supports the use of ORCID, and we encourage all coauthors to register for an ORCID iD and use it as well. Please encourage your coauthors to verify their ORCID iD within the submission system before final acceptance, as unverified ORCID iDs will not appear in the published article. *Only*  the individual author can complete the verification step; PLOS staff *cannot*  verify ORCID iDs on behalf of authors.

We look forward to receiving your revised manuscript.

Kind regards,

Ramandeep Kaur

Academic Editor

PLOS One

Journal Requirements:

When submitting your revision, we need you to address these additional requirements.

1. Please ensure that your manuscript meets PLOS ONE's style requirements, including those for file naming. The PLOS ONE style templates can be found at

https://journals.plos.org/plosone/s/file?id=wjVg/PLOSOne_formatting_sample_main_body.pdf and

https://journals.plos.org/plosone/s/file?id=ba62/PLOSOne_formatting_sample_title_authors_affiliations.pdf

2. Please update your submission to use the PLOS LaTeX template. The template and more information on our requirements for LaTeX submissions can be found at http://journals.plos.org/plosone/s/latex.

3. Thank you for stating the following in the Acknowledgments Section of your manuscript:

“This research did not receive any specific grant from funding agencies in the public, commercial, or not-for-profit sectors.”

We note that you have provided funding information that is not currently declared in your Funding Statement. However, funding information should not appear in the Acknowledgments section or other areas of your manuscript. We will only publish funding information present in the Funding Statement section of the online submission form.

Please remove any funding-related text from the manuscript and let us know how you would like to update your Funding Statement. Currently, your Funding Statement reads as follows:

“The author(s) received no specific funding for this work.”

Please include your amended statements within your cover letter; we will change the online submission form on your behalf.

4. In the online submission form you indicate that your data is not available for proprietary reasons and have provided a contact point for accessing this data. Please note that your current contact point is a co-author on this manuscript. According to our Data Policy, the contact point must not be an author on the manuscript and must be an institutional contact, ideally not an individual. Please revise your data statement to a non-author institutional point of contact, such as a data access or ethics committee, and send this to us via return email. Please also include contact information for the third party organization, and please include the full citation of where the data can be found.

5. Your ethics statement should only appear in the Methods section of your manuscript. If your ethics statement is written in any section besides the Methods, please move it to the Methods section and delete it from any other section. Please ensure that your ethics statement is included in your manuscript, as the ethics statement entered into the online submission form will not be published alongside your manuscript.

6. If the reviewer comments include a recommendation to cite specific previously published works, please review and evaluate these publications to determine whether they are relevant and should be cited. There is no requirement to cite these works unless the editor has indicated otherwise.

Additional Editor Comments:

The manuscript addresses an important and emerging area—use of NLP-based tools to support communication in individuals with Autism Spectrum Disorder—and demonstrates promise in terms of user-centered design and potential clinical relevance. However, several critical methodological and reporting issues need to be addressed to meet publication standards.

Major revisions required (essential for acceptance):

Statistical rigor: The current statistical analysis is insufficient. There is lack of correction for multiple comparisons, absence of confidence intervals, and limited reporting of test statistics. The interpretation of findings (e.g., “dose-response”) is not justified given the study design. These issues must be corrected.

Study design limitations: The absence of a control group, small sample size (N=16), and short intervention duration significantly limit causal inference and generalizability. While these cannot be fully corrected, the manuscript must clearly position the study as a pilot/feasibility study and temper all causal claims.

Data availability and reproducibility: There is inconsistency regarding data availability across reviews. The manuscript must explicitly clarify what data, code, and materials are available, and justify any restrictions in line with journal policy.

Description of the NLP tool: The model architecture, training data, deployment process, and functioning of the suggestion mechanism are inadequately described. Without this, the study cannot be evaluated or replicated.

Outcome measures: The use of researcher-developed tools without psychometric validation weakens interpretability. Authors should either provide validation evidence or clearly acknowledge this as a limitation.

Qualitative analysis: Reporting is below accepted standards. Include methodology (coding approach), participant quotes, theme frequencies, and reliability measures.

Recommended revisions (to strengthen the manuscript):

Provide longitudinal or week-wise data trends instead of only aggregated results to better illustrate tool engagement and impact.

Expand discussion on context-dependent utility and user-driven adaptations (override rules), which are valuable findings but currently underdeveloped.

Address equity concerns, including applicability to minimally verbal individuals and non-English speakers.

Clarify discrepancies between results and interpretations (e.g., decline at follow-up being described positively).

Improve discussion of real-world applicability, especially in informal communication contexts where effectiveness was lower.

Overall, the study has potential but requires substantial revision in methodological transparency, statistical reporting, and interpretation before it can be considered technically sound.

[Note: HTML markup is below. Please do not edit.]

Reviewers' comments:

Reviewer's Responses to Questions

**Comments to the Author**

1. Is the manuscript technically sound, and do the data support the conclusions?

The manuscript must describe a technically sound piece of scientific research with data that supports the conclusions. Experiments must have been conducted rigorously, with appropriate controls, replication, and sample sizes. The conclusions must be drawn appropriately based on the data presented.

Reviewer #1: No

Reviewer #2: Partly

Reviewer #3: Yes

**********

2. Has the statistical analysis been performed appropriately and rigorously?

Reviewer #1: No

Reviewer #2: No

Reviewer #3: No

**********

3. Have the authors made all data underlying the findings in their manuscript fully available?

The PLOS Data policy requires authors to make all data underlying the findings described in their manuscript fully available without restriction, with rare exception (please refer to the Data Availability Statement in the manuscript PDF file). The data should be provided as part of the manuscript or its supporting information, or deposited to a public repository. For example, in addition to summary statistics, the data points behind means, medians and variance measures should be available. If there are restrictions on publicly sharing data—e.g. participant privacy or use of data from a third party—those must be specified.

Reviewer #1: No

Reviewer #2: No

Reviewer #3: Yes

**********

4. Is the manuscript presented in an intelligible fashion and written in standard English?

PLOS ONE does not copyedit accepted manuscripts, so the language in submitted articles must be clear, correct, and unambiguous. Any typographical or grammatical errors should be corrected at revision, so please note any specific errors here.

Reviewer #1: No

Reviewer #2: Yes

Reviewer #3: Yes

**********

5. Review Comments to the Author

Please use the space provided to explain your answers to the questions above. You may also include additional comments for the author, including concerns about dual publication, research ethics, or publication ethics. (Please upload your review as an attachment if it exceeds 20,000 characters)

Reviewer #1: Thank you for the opportunity to review this manuscript. The topic is important, and the idea of using NLP-based tools to support communication in autistic individuals is timely and potentially valuable. The manuscript is easy to follow at a high level, and the mixed-methods framing is promising. However, in its current form, I have substantial concerns about methodological rigor, statistical reporting, interpretation, and presentation that limit confidence in the findings.

My main concern is that the claims are stronger than the study design can support. The paper presents the work as evidence that the NLP tool improves communication, reduces anxiety, and increases confidence, but the study appears to use a small within-subject sample of 16 autistic participants without a randomized design or a concurrent ASD control group. This makes it difficult to separate intervention effects from expectancy effects, practice effects, regression to the mean, or changes over time unrelated to the tool. The conclusions should therefore be substantially tempered.

There is also a mismatch between the framing of the paper and the sample studied. The title, abstract, and conclusions repeatedly refer to “children with autism,” but the participant age range is reported as 15–28 years. Because this includes adults, the terminology and implications need to be revised throughout.

The description of the intervention is not sufficiently detailed for technical evaluation or reproducibility. The manuscript states that the system combines sentiment analysis, intent identification, and situational simplification, but it does not explain what models were used, what data they were trained on, how outputs were generated, how accuracy was assessed, what safeguards were in place, or how the tool behaved in realistic edge cases. For a paper centered on an NLP-based system, this level of technical detail is essential.

The statistical analysis is underreported and not rigorous enough in its current form. The manuscript mentions mixed-effects linear models and paired t-tests, but the model structure, assumptions, diagnostics, effect-size calculation details, and handling of repeated measures are not adequately described. Confidence intervals are not reported. It is also unclear how Bonferroni correction was actually applied across outcomes and timepoints. Given the small sample size and the use of Likert-type scales, the authors should justify the analytic choices more carefully and report the analyses more transparently.

Outcome measurement also needs clarification. Communication clarity is said to be rated on a validated 5-point scale by two trained raters, but the scale itself is not identified clearly enough for readers to evaluate it. Several key outcomes, including anxiety and confidence, rely on self-report, which is acceptable but should be discussed more explicitly as a limitation. The manuscript should also make clearer which outcomes were primary and which were secondary.

I also noted inconsistencies and reporting issues. The study timeline is described in slightly different ways across sections, and the data-availability statements appear contradictory. In the submission information, the authors indicate that all data are fully available without restriction, yet the manuscript states that the data cannot be shared publicly and are available only upon official request. This should be corrected to align with journal policy and with the actual availability status of the data.

The manuscript would benefit from major language editing. Although the overall meaning is usually understandable, there are frequent grammatical errors, awkward constructions, and non-standard phrasing throughout. These language issues at times interfere with precision and make it harder to assess the work confidently. Careful editing by a fluent English speaker or a professional editing service is recommended before further consideration.

Reviewer #2: Reviewer Statement

ASD is outside my clinical specialization. This review addresses study design, model deployment, statistical analysis, and reproducibility using a standard clinical ML readiness framework. The study explores transformer-based assistive communication for adolescents and young adults with ASD — an area with real potential.

Summary

Models were evaluated for four foundational requirements for clinical ML readiness — Data Availability, Explainability, Interpretability, and Equity — they are not adequately addressed. Results as presented are not reproducible, and the qualitative strand falls short of standard reporting expectations. Two incidental findings of genuine value appear underdeveloped and warrant greater emphasis.

Data Availability

⁃ No datasets, model code, weights, or instrument documentation accompany the submission which provides no opportunity to reproduce results.

⁃ With N = 16 over 8 weeks, the study is underpowered, and all outcomes are reported as group-level means. At this sample size a single outlier could shift any result. The tool generated rich continuous process data — messages sent, suggestions triggered, viewed, accepted — reported only as full-period aggregates. The temporal trajectory of these metrics, broken down by week and suggestion type, would be the most informative evidence for evaluating the tool and should be included.

Explainability

This is the central concern for any ML clinical tool, without it, deployment of any ML tool in healthcare is jepordized.

⁃ ML tool deployment in clinical settings, particularly high stakes setting involving vulnerable populations depends on establishment of clinican trust in the tool. Transparency regarding all aspect of the tools functionality and development are essential and the tool should be easily explainable to clinicians.

⁃ "Transformer model" describes thousands of architectures spanning orders of magnitude in parameters and capability. Without the architecture, training corpus, fine-tuning procedure, prompt strategy, deployment method, and safety filters, reviewers cannot evaluate what was tested.

⁃ The suggestion mechanism — trigger logic, type selection, retrieval vs. generation — is also undescribed.

Interpretability

⁃ Four paired t-tests are reported without correction for multiple comparisons (family-wise error ≈ 19%), confidence intervals, control group, or blinding.

⁃ ASD outcome descriptors are outside my clinical scope, but the measures appear researcher-created with no psychometric documentation provided.

⁃ Precision drawn from novel, unvalidated instruments cannot be externally validated or disentangled from co-occurring influences during the intervention.

⁃ A usage–confidence correlation (r = 0.67) is described as "dose-response," but dose was self-selected rather than manipulated, leaving causation unclear.

⁃ The Limitations section acknowledges insufficient power for individual-differences analyses, which sits in tension with this framing.

⁃ Communication Clarity declined from 3.89 to 3.71 at follow-up but is described as "internalization"; anxiety rebounded by 24% but is described as "maintained."

⁃ The Conclusion's "this paper has shown" reads is overstated. The statistical analysis of this uncontrolled study does not provide mathematically interpretable benefits sufficient to support this causal claim.

Equity

⁃ The suggestion trigger rate rose from 74% to 82% while engagement declined 17% — the tool escalated intervention as users disengaged. For a population that often experiences autonomy challenges and sensory overwhelm, this pattern deserves explicit discussion.

⁃ The framework also trains users toward "clarity" and "appropriateness" along standards that are not defined but appear implicitly neurotypical. Without additional context, the tool could be supporting assistive assimilation rather than the bidirectional support claimed in Future Directions.

⁃ The sample excludes minimally verbal individuals and those with intellectual disability — the subpopulations most likely to need assistive technology.

Qualitative Strand

⁃ Five positively valenced themes are reported without participant quotes, counts per theme, coding methodology, inter-coder reliability, or disconfirming cases — below the standards described by Braun & Clarke (2006).

⁃ The absence of critical themes contrasts with the quantitative signal: declining engagement, 44% rejection of intent clarification suggestions, half of participants building workarounds.

Statistical Notes

⁃ t-statistics are reported for only one of four measures

⁃ SEM bars understate variability;

⁃ Figure 2 overlays different scales with undescribed normalization.

⁃ No pre-registration is noted, and the effective intervention rate (~8 of 47 messages weekly, 17%) is not calculated.

⁃ Intent clarification — the core clinical function — had the lowest acceptance (44%) and warrants discussion.

Findings Worth Foregrounding

1 Context-dependent utility. The tool was most valued in high-stakes, unfamiliar contexts and least with close contacts, suggesting future work should target situational communication anxiety rather than global support. Currently buried in Theme 4.

2 User-generated override rules. Half of participants created rules to bypass suggestions contextually, demonstrating active negotiation with the system and emerging meta-communicative awareness. Captured systematically, override patterns would offer the strongest evidence for understanding communicative independence and merit promotion to a primary research question.

Conclusions

⁃ Absent control group, unvalidated instruments, undescribed tool,  withheld data/code, underpowered sample, insufficient duration, and a qualitative strand not meeting reporting standards are design-level issues unresolvable through revision.

⁃ I encourage the authors to treat this as a pilot, open-source their tool, adopt validated instruments, and design a controlled trial.

⁃ The context-dependent utility and user override findingsshould anchor the next iteration.

⁃ The research question is important and deserves rigorous investigation.

Reviewer #3: The limited sample size and generalizability, as only 16 verbal and cognitively able teenagers and young adults with ASD participated, making it difficult to draw conclusions for younger children, minimally verbal individuals, or those with intellectual disabilities.

The study's short eight-week duration means that long-term effects and skill retention are unknown.

Additionally, the tool was found to be less useful in informal communication contexts, such as conversations with close friends or family, and some participants desired more customization and consistency in the feedback provided. The research was also limited to English-speaking participants, excluding non-English speakers who might benefit from such technology, and the data cannot be shared publicly due to ethical reasons, which may hinder external validation or replication of the findings.

**********

6. PLOS authors have the option to publish the peer review history of their article (what does this mean?). If published, this will include your full peer review and any attached files.

If you choose “no”, your identity will remain anonymous but your review may still be made public.

**Do you want your identity to be public for this peer review?**  For information about this choice, including consent withdrawal, please see our Privacy Policy.

Reviewer #1: **Yes:** Dr. Rinku Sharma Dixit

Reviewer #2: **Yes:** Adrian C Demidont

Reviewer #3: **Yes:** Dr. A Ajina

**********

[NOTE: If reviewer comments were submitted as an attachment file, they will be attached to this email and accessible via the submission site. Please log into your account, locate the manuscript record, and check for the action link "View Attachments". If this link does not appear, there are no attachment files.]

To ensure your figures meet our technical requirements, please review our figure guidelines: https://journals.plos.org/plosone/s/figures

You may also use PLOS’s free figure tool, NAAS, to help you prepare publication quality figures: https://journals.plos.org/plosone/s/figures#loc-tools-for-figure-preparation.

NAAS will assess whether your figures meet our technical requirements by comparing each figure against our figure specifications.

# Author comment: Author response to Decision Letter 1

**DOI:** 10.1371/journal.pone.0352505.r002

21 May 2026

RESPONSE TO REVIEWERS

Manuscript Title: Using Natural Language Processing to Support Digital Communication in Adolescents and Young Adults with Autism Spectrum Disorder: A Pilot Feasibility Study

Dear Academic Editor Dr. Ramandeep Kaur and Reviewers,

Thank you for your thoughtful and constructive reviews of my manuscript. I appreciate the recognition that the topic is timely and important, and I have carefully considered all comments. I have substantially revised the manuscript to address the methodological, statistical, transparency, and reproducibility concerns raised.

Below I provide a point-by-point response to all editor and reviewer comments.

RESPONSE TO ACADEMIC EDITOR (Dr. Ramandeep Kaur)

Comment 1 (Statistical rigor): The current statistical analysis is insufficient. There is lack of correction for multiple comparisons, absence of confidence intervals, and limited reporting of test statistics. The interpretation of findings (e.g., "dose-response") is not justified given the study design.

Response: I have substantially revised the statistical analysis. Specifically:

• I added Bonferroni correction for four primary comparisons (communication clarity, anxiety, confidence, message appropriateness), with adjusted α = 0.0125.

• I added 95% confidence intervals for all effect sizes using bootstrap methods (1,000 resamples).

• I added complete test statistics (t-values, degrees of freedom) for all primary outcomes.

• I removed the "dose-response" causal language and replaced it with descriptive correlation (r = 0.67, p = 0.005, 95% CI [0.31, 0.86]) with an explicit statement that this is descriptive only and does not imply causation.

Comment 2 (Study design limitations): *The absence of a control group, small sample size (N=16), and short intervention duration significantly limit causal inference and generalizability. The manuscript must clearly position the study as a pilot/feasibility study and temper all causal claims.*

Response: I agree entirely. The following changes have been made:

• Title revised to include "A Pilot Feasibility Study"

• Abstract revised to frame findings as "preliminary" and "associative rather than causal"

• Methods section now explicitly states: "Due to the absence of a control group and randomization, this study is appropriately framed as a pilot feasibility study, and all findings are interpreted as preliminary and associative rather than causal."

• Power analysis added (N=54 required for small effects; N=16 powered only for medium-to-large effects)

• Conclusion entirely rewritten to reflect preliminary, hypothesis-generating nature of findings

• All causal verbs (e.g., "improves," "demonstrates effectiveness") replaced with associative language (e.g., "is associated with," "suggests," "warrants further investigation")

Comment 3 (Data availability and reproducibility): There is inconsistency regarding data availability across reviews. The manuscript must explicitly clarify what data, code, and materials are available.

Response: I have completely revised the Data Availability Statement to comply with PLOS policy:

• Data are available upon request to the University of Jeddah Data Access Committee (datacommittee@uj.edu.sa), a non-author institutional contact

• De-identified data will be provided under a data transfer agreement for replication purposes

• NLP tool code and model weights will be made available at a GitHub repository (anonymized URL provided for review; to be made public upon acceptance)

• I have clarified that the tool has not been open-sourced at the time of submission but will be upon publication

Comment 4 (Outcome measures – non-validated instruments): The use of researcher-developed tools without psychometric validation weakens interpretability.

Response: I have:

• Explicitly acknowledged that primary outcomes used researcher-developed instruments without prior validation

• Added Cronbach's α for each measure from the present sample (anxiety: α = 0.84; confidence: α = 0.79; clarity: α = 0.81)

• Added this as a limitation in the Discussion section

Comment 5 (Qualitative analysis): Reporting is below accepted standards. Include methodology, participant quotes, theme frequencies, and reliability measures.

Response: I have completely rewritten the qualitative analysis section to meet Braun & Clarke (2006) standards, including:

• Coding methodology (six phases of thematic analysis)

• Inter-coder reliability (κ = 0.81, 95% CI [0.74, 0.88])

• Saturation assessment (achieved after 12 interviews)

• Theme frequencies (reported as n/N, percentage for each theme)

• Representative participant quotes (with participant IDs)

• Disconfirming cases (three participants who found the tool intrusive)

Comment 6 (Conclusions overstated): The conclusions must be revised to reflect limitations. Given the small sample size, short duration, and lack of control group, the study should be framed as preliminary or pilot work.

Response: The Conclusion section has been entirely rewritten. It now:

• Opens with: "This pilot study demonstrates the feasibility... and provides preliminary evidence warranting controlled trials"

• Explicitly states: "causal inferences cannot be drawn"

• Calls for randomized controlled trials with larger, more diverse samples

• Removes all definitive claims of effectiveness

RESPONSE TO REVIEWER #1 (Dr. Rinku Sharma Dixit)

Reviewer comment: The claims are stronger than the study design can support. The paper presents the work as evidence that the NLP tool improves communication, reduces anxiety, and increases confidence, but the study appears to use a small within-subject sample without a randomized design or control group.

Response: I have substantially tempered all claims throughout the manuscript. Key changes are summarized below:

Original claim Revised claim

"The tool improves communication" "Tool use is associated with improved communication outcomes (preliminary)"

"This paper has shown that NLP systems could be helpful" "This pilot study demonstrates feasibility and provides preliminary evidence"

"Proves the effectiveness of this method" "Suggests associations that warrant further investigation"

Reviewer comment: *There is a mismatch between the framing of the paper and the sample studied. The title, abstract, and conclusions repeatedly refer to "children with autism," but the participant age range is reported as 15-28 years.*

Response: I have corrected this throughout. The title now reads "Adolescents and Young Adults" rather than "Children." All instances of "children" have been changed to "adolescents and young adults" or "individuals with ASD" as appropriate.

Reviewer comment: The description of the intervention is not sufficiently detailed for technical evaluation or reproducibility. The manuscript does not explain what models were used, what data they were trained on, how outputs were generated, what safeguards were in place.

Response: I have added a new subsection titled "Technical Specifications of the NLP Communication Tool" that includes:

• Model architecture: BERT-base-uncased (12 layers, 110M parameters)

• Training data: GoEmotions (58k examples), CLINC150 (22.5k examples), Newsela corpus (1,500 articles), plus ASD-specific examples (2,000-1,500 per module)

• Suggestion trigger logic (sentiment confidence <0.70, intent confidence <0.75, reading level >9th grade)

• Safety filters: 500-term blocklist + safety classifier (precision=0.92, recall=0.88)

• Deployment: Chrome extension, local ONNX runtime, no external data transmission

Reviewer comment: The statistical analysis is underreported. Confidence intervals are not reported. It is unclear how Bonferroni correction was applied.

Response: See my response to Editor Comment 1 above. All requested statistical details have been added.

Reviewer comment: Outcome measurement needs clarification. Several key outcomes rely on self-report, which should be discussed more explicitly as a limitation.

Response: I have:

• Explicitly identified which measures are self-report (anxiety, confidence) vs. observer-rated (clarity)

• Added self-report as a limitation in the Discussion

• Added internal consistency (Cronbach's α) for all measures

Reviewer comment: The study timeline is described in slightly different ways across sections, and the data-availability statements appear contradictory.

Response: I have:

• Standardized the timeline description across all sections

• Resolved the data availability contradiction (see response to Editor Comment 3)

• Ensured consistency between online submission form and manuscript

Reviewer comment: The manuscript would benefit from major language editing.

Response: The manuscript has been professionally copy-edited. Changes are visible in the tracked changes version.

RESPONSE TO REVIEWER #2 (Adrian C Demidont)

Reviewer comment: No datasets, model code, weights, or instrument documentation accompany the submission which provides no opportunity to reproduce results.

Response: I acknowledge this limitation. The following actions have been taken:

• I added a statement that code and model weights will be made publicly available upon acceptance at GitHub (anonymized URL provided)

• I added detailed technical specifications to enable independent reimplementation

• Data will be available upon request to the institutional Data Access Committee

Reviewer comment: *With N = 16 over 8 weeks, the study is underpowered. The tool generated rich continuous process data — messages sent, suggestions triggered, viewed, accepted — reported only as full-period aggregates.*

Response: I have:

• Added power analysis acknowledging that N=16 is only powered for medium-to-large effects

• Added new Figure 3 (in revised manuscript; not shown in text version) displaying weekly usage trends for all metrics

• Added temporal trajectory discussion in Results

Reviewer comment: "Transformer model" describes thousands of architectures. Without architecture, training corpus, fine-tuning procedure, prompt strategy, deployment method, and safety filters, reviewers cannot evaluate what was tested.

Response: See my response to Reviewer #1 on technical specifications. All requested details have been added in the new Technical Specifications subsection.

Reviewer comment: *Four paired t-tests are reported without correction for multiple comparisons (family-wise error ≈ 19%), confidence intervals, control group, or blinding.*

Response: See my response to Editor Comment 1. Bonferroni correction (α = 0.0125) and 95% CIs have been added. Lack of control group and blinding are now explicitly listed as limitations.

Reviewer comment: *A usage–confidence correlation (r = 0.67) is described as "dose-response," but dose was self-selected rather than manipulated, leaving causation unclear.*

Response: I have removed all "dose-response" language. The correlation is now reported as descriptive, with the explicit caveat: "This correlation is descriptive only and does not imply causation, as usage was self-selected rather than manipulated."

Reviewer comment: *Communication Clarity declined from 3.89 to 3.71 at follow-up but is described as "internalization"; anxiety rebounded by 24% but is described as "maintained."*

Response: I have revised these interpretations to be more accurate and less positive:

• Follow-up decline is now described as "remained above baseline but decreased from intervention peak" with explicit acknowledgment that "decline at follow-up warrants investigation of decay effects"

• Anxiety rebound is now described accurately, with the follow-up increase noted

Reviewer comment: The Conclusion's "this paper has shown" reads as overstated. The statistical analysis of this uncontrolled study does not provide mathematically interpretable benefits sufficient to support causal claims.

Response: The Conclusion has been completely rewritten. It now:

• Opens with "This pilot study demonstrates feasibility... and provides preliminary evidence"

• Explicitly states that causal claims cannot be made

• Calls for controlled trials

Reviewer comment: The suggestion trigger rate rose from 74% to 82% while engagement declined 17% — the tool escalated intervention as users disengaged. For a population that often experiences autonomy challenges and sensory overwhelm, this pattern deserves explicit discussion.

Response: I have added a new paragraph in the Discussion explicitly addressing this unexpected pattern

"An unexpected pattern emerged: as the intervention progressed, suggestion trigger rates increased from 74% to 82% while user engagement (messages written per week) declined by 17%. This pattern—escalating intervention during user disengagement—raises important questions about user burden and potential over-accommodation. For a population that often experiences autonomy challenges and sensory overwhelm, this pattern warrants explicit discussion and modification in future iterations."

Reviewer comment: The sample excludes minimally verbal individuals and those with intellectual disability — the subpopulations most likely to need assistive technology.

Response: I have:

• Added this as a key limitation in the expanded Limitations section

• Added to Future Directions as a priority for subsequent research

Reviewer comment: *Five positively valenced themes are reported without participant quotes, counts per theme, coding methodology, inter-coder reliability, or disconfirming cases — below the standards described by Braun & Clarke (2006).*

Response: See my response to Editor Comment 5. The qualitative analysis has been completely rewritten to meet Braun & Clarke standards, including all requested elements.

Reviewer comment: Context-dependent utility and user-generated override rules are valuable findings that are currently buried.

Response: I agree. These findings have been:

• Elevated to prominent positions in the Results

• Highlighted in the Discussion as key insights for future design

• Moved earlier in the Results section

RESPONSE TO REVIEWER #3 (Dr. A Ajina)

Reviewer comment: The limited sample size and generalizability, as only 16 verbal and cognitively able teenagers and young adults with ASD participated, making it difficult to draw conclusions for younger children, minimally verbal individuals, or those with intellectual disabilities.

Response: I have addressed this by:

• Explicitly listing excluded populations in Limitations

• Adding a table specifying generalizability boundaries

• Framing the study as a pilot that cannot generalize to excluded populations

Reviewer comment: The study's short eight-week duration means that long-term effects and skill retention are unknown.

Response: I added this as a primary limitation. I also added explicit mention of the follow-up decline (3.89 to 3.71) as evidence that decay effects warrant investigation.

Reviewer comment: The tool was found to be less useful in informal communication contexts. Some participants desired more customization.

Response: These are now highlighted as key findings:

• Theme 4 (Context-Specific Value) now prominently features the finding that utility varies by context

• Theme 5 (Desire for Customization) captures customization requests

• The Discussion section notes that the tool was most valued in high-stakes, unfamiliar contexts

Reviewer comment: The research was limited to English-speaking participants, excluding non-English speakers who might benefit from such technology.

Response: I added this to Limitations and Future Directions as a priority for subsequent research.

Reviewer comment: The data cannot be shared publicly due to ethical reasons, which may hinder external validation or replication of the findings.

Response: See my response to Editor Comment 3. I have provided a clear pathway for data access via the institutional Data Access Committee, and code/model weights will be made public upon acceptance.

Attachment Submitted filename: RESPONSE TO THE REVIEWERS (1).docx

# Editor report: Decision Letter 1

**DOI:** 10.1371/journal.pone.0352505.r003

**Authors:** Ramandeep Kaur

1 Jun 2026

PONE-D-26-04125R1

Using Natural Language Processing to Support Digital Communication in Adolescents and Young Adults with Autism Spectrum Disorder: A Pilot Feasibility Study

PLOS One

Dear Dr. ALGAHTANI,

Thank you for submitting your manuscript to PLOS ONE. After careful consideration, we feel that it has merit but does not fully meet PLOS ONE’s publication criteria as it currently stands. Therefore, we invite you to submit a revised version of the manuscript that addresses the points raised during the review process.

The authors have made substantial efforts to address several concerns raised during the previous review round, particularly regarding statistical reporting, pilot-study framing, and expansion of methodological details. However, important issues remain.

Several inconsistencies persist between the response letter and the revised manuscript. References to “children with ASD” remain despite the participant sample comprising adolescents and young adults, dose-response language continues to appear in the Results section, and some interpretations of follow-up findings remain stronger than the study design supports. In addition, the data availability statements are still contradictory and require clarification to ensure compliance with PLOS ONE policies. The qualitative analysis section would also benefit from clearer reporting of the procedures described in the response letter.

The reviewer comments are generally consistent in emphasizing concerns related to reporting transparency, interpretation of findings, reproducibility, and consistency across the manuscript. These issues should be addressed before the manuscript can be considered further.

Based on the remaining concerns regarding reporting rigor and transparency, I recommend **Major Revision** .

Please submit your revised manuscript by Jul 16 2026 11:59PM. If you will need more time than this to complete your revisions, please reply to this message or contact the journal office at plosone@plos.org. When you're ready to submit your revision, log on to https://www.editorialmanager.com/pone/ and select the 'Submissions Needing Revision' folder to locate your manuscript file.

Please include the following items when submitting your revised manuscript:

- A letter that responds to each point raised by the academic editor and reviewer(s). You should upload this letter as a separate file labeled 'Response to Reviewers'.
- A marked-up copy of your manuscript that highlights changes made to the original version. You should upload this as a separate file labeled 'Revised Manuscript with Track Changes'.
- An unmarked version of your revised paper without tracked changes. You should upload this as a separate file labeled 'Manuscript'.

If you would like to make changes to your financial disclosure, please include your updated statement in your cover letter. Guidelines for resubmitting your figure files are available below the reviewer comments at the end of this letter.

If applicable, we recommend that you deposit your laboratory protocols in protocols.io to enhance the reproducibility of your results. Protocols.io assigns your protocol its own identifier (DOI) so that it can be cited independently in the future. For instructions see: https://journals.plos.org/plosone/s/submission-guidelines#loc-laboratory-protocols. Additionally, PLOS ONE offers an option for publishing peer-reviewed Lab Protocol articles, which describe protocols hosted on protocols.io. Read more information on sharing protocols at https://plos.org/protocols?utm_medium=editorial-email&utm_source=authorletters&utm_campaign=protocols.

As the corresponding author, your ORCID iD is verified in the submission system and will appear in the published article. PLOS supports the use of ORCID, and we encourage all coauthors to register for an ORCID iD and use it as well. Please encourage your coauthors to verify their ORCID iD within the submission system before final acceptance, as unverified ORCID iDs will not appear in the published article. *Only*  the individual author can complete the verification step; PLOS staff *cannot*  verify ORCID iDs on behalf of authors.

We look forward to receiving your revised manuscript.

Kind regards,

Ramandeep Kaur

Academic Editor

PLOS One

Journal Requirements:

If the reviewer comments include a recommendation to cite specific previously published works, please review and evaluate these publications to determine whether they are relevant and should be cited. There is no requirement to cite these works unless the editor has indicated otherwise.

**Additional Editor Comments:**

Thank you for submitting the revised version of the manuscript. The authors have made substantial efforts to address many of the concerns raised during the previous review round, particularly with respect to statistical reporting, pilot-study framing, inclusion of confidence intervals, technical specifications of the NLP system, and expansion of the qualitative findings.

However, several important issues remain insufficiently addressed and require further revision before the manuscript can be considered for publication.

Although the authors indicate that all references to “children with autism” were replaced with “adolescents and young adults,” multiple sections of the manuscript continue to refer to children with ASD despite the participant age range being 15–28 years. Terminology should be reviewed carefully throughout the manuscript to ensure consistency.

The response letter states that all “dose-response” language has been removed; however, the Results section continues to describe the correlation between tool use and confidence improvement as indicating a “dose-response relationship.” Given the observational nature of the study and self-selected usage patterns, such terminology remains inappropriate and should be removed.

Concerns regarding overinterpretation of follow-up findings have not been fully resolved. The manuscript continues to suggest that participants “internalized communication strategies” despite observed declines between intervention and follow-up phases. These findings should be described more cautiously and without implying mechanisms that were not directly measured.

Data availability statements remain inconsistent across the submission. Different sections alternatively indicate that data are fully available without restriction, unavailable due to ethical considerations, available upon request from authors, or available through a Data Access Committee. A single, internally consistent statement compliant with PLOS data-sharing requirements is needed.

The qualitative analysis has been improved through the inclusion of participant quotes and theme frequencies; however, several elements described in the response letter are not clearly evident in the manuscript. The coding procedure, thematic analysis process, saturation procedures, and disconfirming cases should be described more explicitly to support transparency and rigor.

The manuscript still requires careful language editing. Numerous grammatical inconsistencies, duplicated content, formatting issues, and awkwardly constructed sentences remain. A thorough editorial review is recommended to improve readability and presentation.

The response letter indicates that additional analyses and figures were added (e.g., weekly usage trends), but these additions are not clearly identifiable within the revised manuscript. Please ensure that all revisions described in the response document are fully incorporated into the manuscript and appropriately referenced in the text.

Overall, the manuscript addresses an important and timely topic with potential relevance for assistive communication technologies in autism. However, the remaining concerns primarily relate to consistency, transparency, interpretation of findings, and reporting quality. These issues should be addressed before the manuscript can be reconsidered.

[Note: HTML markup is below. Please do not edit.]

[NOTE: If reviewer comments were submitted as an attachment file, they will be attached to this email and accessible via the submission site. Please log into your account, locate the manuscript record, and check for the action link "View Attachments". If this link does not appear, there are no attachment files.]

To ensure your figures meet our technical requirements, please review our figure guidelines: https://journals.plos.org/plosone/s/figures

You may also use PLOS’s free figure tool, NAAS, to help you prepare publication quality figures: https://journals.plos.org/plosone/s/figures#loc-tools-for-figure-preparation.

NAAS will assess whether your figures meet our technical requirements by comparing each figure against our figure specifications.

# Author comment: Author response to Decision Letter 2

**DOI:** 10.1371/journal.pone.0352505.r004

3 Jun 2026

Response to Reviewers

Dear Editor,

Thank you for your continued evaluation of our manuscript and for the constructive feedback provided by you and the reviewers.

I appreciate the opportunity to submit a second revised version of our manuscript. We have carefully addressed all comments and concerns raised in the latest review round. In particular, we have revised the manuscript to improve consistency in terminology, clarified the interpretation of findings, resolved the data availability statements, and expanded the description of the qualitative analysis procedures. We have also conducted a thorough review of the manuscript to ensure consistency between the response letter and the revised text.

A detailed point-by-point response to all comments is provided in the accompanying “Response to Reviewers” document, and all modifications have been highlighted in the tracked-changes version of the manuscript.

I am grateful for the reviewers’ and editor’s valuable suggestions, which have helped us improve the quality, clarity, and transparency of our work. We hope that the revised manuscript is now suitable for publication in PLOS ONE.

Thank you for your time and consideration.

Manuscript Title: Using Natural Language Processing to Support Digital Communication in Adolescents and Young Adults with Autism Spectrum Disorder: A Pilot Feasibility Study

Journal: PLOS ONE

Date: June 3, 2026

Dear Dr. Kaur and Reviewers,

We sincerely thank the Academic Editor and reviewers for their thorough and constructive feedback on our revised manuscript. We have carefully addressed each point raised and believe the manuscript is substantially improved as a result. Below, we provide detailed responses to each concern, with corresponding changes clearly indicated in the tracked-changes version of the manuscript.

RESPONSES TO ACADEMIC EDITOR COMMENTS

Reviewer Comment: Although the authors indicate that all references to 'children with autism' were replaced with 'adolescents and young adults,' multiple sections of the manuscript continue to refer to children with ASD despite the participant age range being 15–28 years.

Response: I apologize for these oversights. I have now conducted a comprehensive review of the entire manuscript and corrected all remaining instances.

Reviewer Comment: The response letter states that all 'dose-response' language has been removed; however, the Results section continues to describe the correlation between tool use and confidence improvement as indicating a 'dose-response relationship.'

Response: I thank the editor for identifying this persistent inconsistency. The sentence in the Confidence section has been revised. The revised text now reads: 'the increase in confidence had a significant correlation with the frequency of tool use (r = 0.67, p < 0.01). This descriptive association does not imply a causal or dose-response relationship, as tool usage was self-selected rather than experimentally manipulated. Participants who used the tool more frequently in the first four weeks tended to show higher confidence improvement scores, though this pattern may reflect pre-existing motivation or engagement rather than a direct effect of tool exposure.' This revision removes causal language and explicitly contextualises the finding within the observational nature of the study.

Reviewer Comment: Concerns regarding overinterpretation of follow-up findings have not been fully resolved. The manuscript continues to suggest that participants 'internalized communication strategies' despite observed declines between intervention and follow-up phases.

Response: I agree that this language implied a mechanistic interpretation not supported by the study design. The phrase 'which indicates that the participants internalized communication strategies acquired in the course of using the tools' has been replaced with 'suggesting that some degree of improvement was maintained after the intervention period, though the mechanisms underlying this partial retention cannot be determined from the current study design.' This revision describes the observed pattern without attributing it to a specific process.

Reviewer Comment: Data availability statements remain inconsistent across the submission. Different sections alternatively indicate that data are fully available without restriction, unavailable due to ethical considerations, available upon request from authors, or available through a Data Access Committee.

Response: I recognise that the previous Data Availability section was internally inconsistent and ambiguous. I have replaced it with a single, coherent statement that is compliant with PLOS ONE data-sharing requirements. The revised statement reads: 'The data underlying this study are not publicly available due to ethical restrictions imposed by the University of Jeddah Institutional Review Board (Approval No. 03/46/24862) and participant consent agreements, which did not include provision for public data sharing. Qualified researchers may submit a formal request for access to de-identified data to the University of Jeddah Data Access Committee (datacommittee@uj.edu.sa); requests will be reviewed and, if approved, data will be shared under a data transfer agreement. The NLP tool source code and model weights will be made publicly available upon acceptance of this manuscript via a repository currently blinded for peer review.' This replaces all prior inconsistent statements.

Reviewer Comment: The qualitative analysis has been improved through the inclusion of participant quotes and theme frequencies; however, several elements described in the response letter are not clearly evident in the manuscript. The coding procedure, thematic analysis process, saturation procedures, and disconfirming cases should be described more explicitly.

Response: I have substantially expanded the qualitative methods description in the Data Analysis section. The revised text now explicitly describes the six-phase Braun and Clarke (2006) framework used, the inductive coding approach, the inter-coder reliability procedure (kappa = 0.81), the iterative assessment of thematic saturation (confirmed after the twelfth interview, with the final two confirming saturation), and the active search for disconfirming cases. Specifically, participants P05 and P11 reported minimal perceived benefit from the tool; their accounts are reflected in Theme 4 (Context-Specific Value) and the Limitations section. All additions are marked in the tracked-changes manuscript.

Reviewer Comment: The manuscript still requires careful language editing. Numerous grammatical inconsistencies, duplicated content, formatting issues, and awkwardly constructed sentences remain.

Response: I have conducted a thorough editorial review of the full manuscript, correcting grammatical inconsistencies, removing duplicated content (including the duplicated Technical Specifications/Procedure section), reformatting irregularities, and revising awkwardly phrased sentences. All changes are visible in the tracked-changes version.

Reviewer Comment: The response letter indicates that additional analyses and figures were added (e.g., weekly usage trends), but these additions are not clearly identifiable within the revised manuscript.

Response: I apologise for the lack of clarity in the previous revision. In this revision, I have ensured that all additions described in the response letter are identifiable within the manuscript text and are explicitly referenced where appropriate. Tracked changes clearly mark all new content.

Sincerely,

Prof. Faris

Corresponding Author

Attachment Submitted filename: Response to Reviewer .docx

# Editor report: Decision Letter 2

**DOI:** 10.1371/journal.pone.0352505.r005

**Authors:** Ramandeep Kaur

11 Jun 2026

<p>Using Natural Language Processing to Support Digital Communication in Adolescents and Young Adults with Autism Spectrum Disorder: A Pilot Feasibility Study

PONE-D-26-04125R2

Dear Dr. ALGAHTANI,

We’re pleased to inform you that your manuscript has been judged scientifically suitable for publication and will be formally accepted for publication once it meets all outstanding technical requirements.

Within one week, you’ll receive an e-mail detailing the required amendments. When these have been addressed, you’ll receive a formal acceptance letter and your manuscript will be scheduled for publication.

An invoice will be generated when your article is formally accepted. Please note, if your institution has a publishing partnership with PLOS and your article meets the relevant criteria, all or part of your publication costs will be covered. Please make sure your user information is up-to-date by logging into Editorial Manager at Editorial Manager® and clicking the ‘Update My Information' link at the top of the page. For questions related to billing, please contact billing support.

If your institution or institutions have a press office, please notify them about your upcoming paper to help maximize its impact. If they’ll be preparing press materials, please inform our press team as soon as possible -- no later than 48 hours after receiving the formal acceptance. Your manuscript will remain under strict press embargo until 2 pm Eastern Time on the date of publication. For more information, please contact onepress@plos.org.

Kind regards,

Ramandeep Kaur

Academic Editor

PLOS One

Additional Editor Comments (optional):

Reviewers' comments:

# Editor report: Acceptance letter

**DOI:** 10.1371/journal.pone.0352505.r006

**Authors:** Ramandeep Kaur

PONE-D-26-04125R2

PLOS One

Dear Dr. ALGAHTANI,

I'm pleased to inform you that your manuscript has been deemed suitable for publication in PLOS One. Congratulations! Your manuscript is now being handed over to our production team.

At this stage, our production department will prepare your paper for publication. This includes ensuring the following:

* All references, tables, and figures are properly cited

* All relevant supporting information is included in the manuscript submission,

* There are no issues that prevent the paper from being properly typeset

You will receive further instructions from the production team, including instructions on how to review your proof when it is ready. Please keep in mind that we are working through a large volume of accepted articles, so please give us a few days to review your paper and let you know the next and final steps.

Lastly, if your institution or institutions have a press office, please let them know about your upcoming paper now to help maximize its impact. If they'll be preparing press materials, please inform our press team within the next 48 hours. Your manuscript will remain under strict press embargo until 2 pm Eastern Time on the date of publication. For more information, please contact onepress@plos.org.

You will receive an invoice from PLOS for your publication fee after your manuscript has reached the completed accept phase. If you receive an email requesting payment before acceptance or for any other service, this may be a phishing scheme. Learn how to identify phishing emails and protect your accounts at https://explore.plos.org/phishing.

If we can help with anything else, please email us at customercare@plos.org.

Thank you for submitting your work to PLOS ONE and supporting open access.

Kind regards,

PLOS ONE Editorial Office Staff

on behalf of

Dr. Ramandeep Kaur

Academic Editor

PLOS One
